# Physiological and Transcriptomic Analyses Unveil the Preservation Mechanism of *Streptomyces albulus* Ah11601 Fermentation Broth on ‘Shine Muscat’ Grapes

**DOI:** 10.3390/genes16040468

**Published:** 2025-04-19

**Authors:** Chao-Tian Lv, Huan Li, Ri-Mao Hua

**Affiliations:** 1 Key Lab of Agri-Food Safety of Anhui Province, College of Resource and Environmental Science, Anhui Agricultural University, Hefei 230036, China; 2 College of Food and Bio-Engineering, Bengbu University, Bengbu 233030, China; huanli@bbc.edu.cn

**Keywords:** ‘Shine Muscat’ grape, postharvest preservation, transcriptome, antioxidant system, secondary metabolic pathway, hormone signaling

## Abstract

Background/Objectives: Grapes (*Vitis vinifera*), particularly ‘Shine Muscat’, are prone to postharvest quality loss mainly due to poor storage tolerance. Actinomycetes are microbial resources that produce secondary metabolites that exhibit notable functional properties. Methods: This study explored the use of *Streptomyces albulus* Ah11601 fermentation broth (SFB) as a postharvest treatment to preserve ‘Shine Muscat’ grape quality during 6 days of room temperature storage using physiological, transcriptomic, and bioinformatics analyses to elucidate the underlying regulatory mechanism. Results: The results demonstrated that, compared to the control group stored at room temperature (25 °C) for 6 days (6D), the SFB-treated group (T6D) presented a significant delay in the decrease in fruit hardness and vitamin C content. Further investigations revealed that the 6D treatment significantly elevated lipoxygenase activity, MDA content, O_2_^−^ generation rate, and H_2_O_2_ levels. In addition, both the 6D and T6D treatments significantly increased the activities of SOD and APX. Functional enrichment analysis revealed that the upregulated DEGs in the 6D group were predominantly enriched in pathways such as phenylpropanoid biosynthesis; flavonoid biosynthesis; phenylalanine metabolism; and stilbenoid, diarylheptanoid, and gingerol biosynthesis. The downregulated DEGs were enriched primarily in the endoplasmic reticulum protein processing pathway. In the T6D group, the upregulated DEGs were predominantly enriched in the zeatin biosynthesis pathway. In addition, significant alterations in the expression of genes associated with the ethylene and abscisic acid signaling pathways were detected. Conclusions: In conclusion, SFB treatment effectively mitigated the deterioration of the postharvest quality of ‘Shine Muscat’ grapes by preserving the cellular redox balance, regulating cytokinin and ethylene biosynthesis, and optimizing the regulation of ethylene and abscisic acid signaling.

## 1. Introduction

With the continuous development of the economy and society and improvements in living standards, consumer demands for food have increased, with a growing emphasis on convenience, speed, safety, and health-conscious dietary practices. A critical challenge in the food industry is the preservation and extension of shelf life. Grapes (*V. vinifera* L.), perennial deciduous vines, are rich in various nutrients, including proteins, vitamins, amino acids, and minerals. They are widely recognized for their anti-inflammatory, anticancer, antioxidant, and cardiovascular protective properties, making them highly popular among consumers [1]. The ‘Shine Muscat’ grape, a hybrid cultivar derived from Akitsu-21 and Hakunan, presents a yellow–green color, thin skin, juicy flesh, small seeds, and a distinct rose fragrance [2]. However, during postharvest storage, the fruit is prone to issues such as dropping, decay, browning, and softening. This leads to a decline in fruit quality and a significant reduction in shelf life. Therefore, maintaining the firmness and quality of ‘Shine Muscat’ grapes and delaying their deterioration during storage are of paramount importance.

Research on fruit and vegetable preservation primarily includes methods such as preservatives, modified atmosphere packaging, irradiation, low-temperature storage, and electric field preservation [3,4,5,6,7]. These techniques generally work by reducing respiration, inhibiting pathogen growth, and eliminating pathogens, thereby extending shelf life and maintaining quality. Recent studies have indicated that storage at 4 °C extends grape shelf life by slowing physiological deterioration and improving flavor, with optimal consumption within 4 days. Conversely, 25 °C storage increases phenolic and flavonoid compounds, which are linked to plant stress responses [3]. Zhao et al. [4] demonstrated that near-ice temperature storage effectively prolongs sweet cherry preservation by increasing fruit quality parameters, including firmness and color retention, while significantly increasing ascorbic acid content. This storage condition concurrently reduces oxidative stress through suppressed ROS accumulation and membrane lipid peroxidation, consequently enhancing antioxidant capacity. Sulfur dioxide (SO_2_) extends fresh grape shelf life by reducing cell wall degradation, enhancing peel defense, activating secondary metabolism, inhibiting ethylene signaling, and delaying softening and senescence [5]. Antibacterial nanocomposites of chitosan, cinnamon oil, and titanium dioxide (TiO_2_) enhanced strawberry firmness, reduced weight loss and mold, and extended shelf life by disrupting bacterial cell walls [7]. Additionally, the application of calcium propionate during the fruit development stage of ‘Shine Muscat’ grapes effectively controls both pre- and postharvest diseases and extends shelf life [8]. In summary, physical, chemical, and plant extract-based methods are effective for postharvest fruit preservation. However, owing to their wide availability, robust adaptability, rapid propagation rates, and substantial antimicrobial efficacy, biological preservation technologies have garnered increasing interest in recent years. These technologies predominantly rely on antagonistic microorganisms or metabolic products to inhibit or eradicate pathogens on the surface of fruits and vegetables, thereby achieving preservation [9]. Research by Zhong et al. [10] on the fermentation liquid of *Streptomyces* 702 revealed that it retards water evaporation from fruits; inhibits the growth of harmful bacteria; and provides effective preservation for apples, pears, kiwis, and melons. Tian et al. [11] demonstrated that two antagonistic microorganisms, *Cryptococcus laurentii* and *Rhodotorula glutinis*, can reduce the incidence of disease in sweet cherries (*Prunus avium* L. cv. Hongdeng), and *C*. *albidus* (Saito) Skinner has also been shown to inhibit *Botrytis cinerea* and *Penicillium expansum* in apples, offering preservation benefits [12]. Despite the significant progress made in the application of biological preservation technologies for postharvest fruit and vegetable storage, further in-depth investigations are warranted to explore their potential and underlying mechanisms in extending the shelf life of ‘Shine Muscat’ grapes.

Transcriptomics employs high-throughput sequencing to analyze cellular gene expression profiles, revealing RNA-level gene–phenotype relationships, transcriptional variations, and functional genes, thus advancing the understanding of gene regulation. Transcriptomics has been extensively applied in postharvest preservation research on fruits and vegetables. For instance, Li et al. [13] demonstrated that *Bacillus velezensis* HY19 fermentation broth enhances citrus preservation by suppressing the expression of genes related to N-glycan and protein biosynthesis in *Penicillium digitatum*, improving antioxidant activity and disease resistance, and reducing fungal infections. Additionally, Wang et al. [14] employed an integrated transcriptomic and proteomic approach to investigate the antifungal mechanisms of volatile organic compounds (VOCs) produced by *Bacillus subtilis* CF-3 against *Colletotrichum gloeosporioides*. This study revealed that VOCs significantly downregulated the expression of genes associated with critical fungal processes, including cell membrane fluidity, cell wall integrity, energy metabolism, and cell wall-degrading enzyme production. Notably, key differentially expressed genes (DEGs) and proteins (DEPs) involved in ergosterol biosynthesis and unsaturated fatty acid biosynthesis were most profoundly affected. These findings provide a theoretical foundation for the potential application of *B. subtilis* CF-3 in the postharvest protection of fruits and vegetables. Consequently, RNA sequencing has emerged as a powerful technological platform for elucidating the molecular mechanisms underlying postharvest preservation at the transcriptional level.

Actinobacteria are ubiquitously distributed in natural environments, including soil and oceans, and can even be found in extreme habitats, such as the atmosphere and deserts, with soil serving as a primary source. As a crucial group of microorganisms, actinobacteria produce a diverse array of secondary metabolites that exhibit remarkable biological activities, including antibacterial, anti-inflammatory, insecticidal, and herbicidal properties. Accumulating evidence has demonstrated the efficacy of actinobacterial metabolites in suppressing postharvest pathogenic proliferation in fruits and vegetables [10,11,13,14]. However, current mechanistic investigations remain predominantly confined to phenotypic physiological and biochemical assessments, with limited ability to elucidate the underlying molecular pathways at the omics level. Notably, the ‘Shine Muscat’ grape, as a premium table grape cultivar, faces significant challenges in maintaining postharvest quality. While transcriptomic profiling has been established for postharvest treatments, including 1-methylcyclopropene (1-MCP) [15,16], sulfur dioxide (SO_2_) [15,16], and methyl jasmonate (MeJA) [17], the molecular regulatory effects on this cultivar based on the transcriptome of the fermentation broth of Actinobacteria remain unexplored. To address this research deficiency, we recently isolated an Actinobacterium, *S. albulus* Ah11601, with potent antibacterial activity from the soil of a forest in Huangcangyu, Anhui Province, China (117°04′ N, 34°03′ E). On the basis of these findings, we hypothesize that treatment with *S. albulus* Ah11601 fermentation broth may increase the postharvest quality of ‘Shine Muscat’ grapes. To test this hypothesis, this study employs a novel application of actinobacterial fermentation broth derived from unique habitats for table grape storage, combining physiological, biochemical, and transcriptomic analyses. Collectively, our results elucidate the mechanism underlying actinobacterial fermentation broth-mediated fruit quality modulation and establish a molecular framework for the development of novel biopreservatives for postharvest management.

## 2. Materials and Methods

### 2.1. Grape Samples and Treatments

The type strain *S. albulus* Ah11601 (deposited at the China General Microbiological Culture Collection Center with accession No. 11363) was screened from the soil of a forest in Huang-cangyu, Anhui Province, China (117°04′ N, 34°03′ E). To produce fermentation broth, the liquid culture medium for *S. albulus* Ah11601 was formulated with the following composition (g·L^−1^): soluble starch (30 g), soybean meal (10 g), KH_2_PO_4_ (2.5 g), MgSO_4_·7H_2_O (1.5 g), NaCl (1.0 g), ZnCl_2_ (0.03 g), and FeSO_4_·7H_2_O (0.02 g). Fermentation parameters were optimized and maintained under controlled conditions: incubation temperature at 27 °C, pH adjusted to 6.0, orbital shaking at 160 rpm, and an inoculum volume of 10% (*v*/*v*), with a total fermentation period of 168 h. After fermentation, the culture broth was processed through ultrafiltration under aseptic conditions, and the clarified filtrate was diluted 100-fold and stored at 4 °C in sterile containers for subsequent experimental applications.

The ‘Shine Muscat’ grapes were harvested from a local vineyard during the commercial ripening stage, with rigorous selection criteria applied to ensure homogeneity in maturity indices, dimensional uniformity, and the exclusion of mechanically damaged or pathogen-infected samples. Following harvest, the grape clusters were immediately transported under controlled conditions to the laboratory facility within one hour [18]. The experimental design included three distinct treatment groups: (1) an untreated control group (CK); (2) a group subjected to room temperature storage (25 °C) for a duration of 6 days (6D); and (3) a group treated with a diluted fermentation broth of *S. albulus* followed by identical room temperature storage (25 °C) for a duration of 6 days (T6D). For comprehensive analysis, freshly harvested samples were partitioned for dual processing: one subset was allocated for immediate assessment of physiological and biochemical parameters, whereas a parallel subset was rapidly cryopreserved in liquid nitrogen to ensure RNA integrity for subsequent transcriptomic profiling and differential gene expression analysis.

### 2.2. Determination of Hardness and Vitamin C Content

Fruit hardness was assessed using a GY-4 hardness tester (Tuopu Instrument, Ningbo, China) featuring a 10 mm probe [19]. A 1 cm^2^ section of the peel was excised from the equatorial area opposite the stem, and the probe was inserted into the flesh to a depth of 10 mm. Measurements were conducted in at least six replicates per group, with results recorded in kg·cm^−2^.

The vitamin C content was measured according to the methods of Wang et al. [20] with modifications. Fresh grape samples (0.5 g) were homogenized in 20 mL of 1% oxalic acid, filtered through gauze, and centrifuged. A 10 mL filtrate aliquot was titrated with 0.1% 2,6-dichlorophenolindophenol sodium solution until a stable pink endpoint was reached (≥15 s). The titrant volume was recorded, and the vitamin C content was calculated accordingly.

### 2.3. Determination of Malondialdehyde and Hydrogen Peroxide Content and the Superoxide Radical Production Rate

The malondialdehyde (MDA) content was measured using the thiobarbituric acid (TBA) method [21]. Briefly, 0.2 g of grape fruit was homogenized in 5 mL of 10% trichloroacetic acid (TCA) on ice and centrifuged at 10,000× *g* for 20 min. A 2 mL aliquot of the supernatant was mixed with 2 mL of 10% TCA containing 0.6% TBA, incubated at 95 °C for 15 min, and cooled on ice. After centrifugation at 10,000 rpm (4 °C, 5 min), the MDA content was calculated from the absorbance values at 450 nm, 532 nm, and 600 nm.

The superoxide radical (O_2_^−^) generation rate was quantified as follows: 0.2 g of grape fruit tissue was homogenized in 1 mL of ice-cold 50 mM phosphate buffer (pH 7.8) and centrifuged at 12,000× *g* for 20 min at 4 °C. The supernatant was incubated with 0.5 mL of phosphate buffer and 1.5 mL of 1 mM hydroxylamine hydrochloride at 25 °C for 1 h. Then, 2 mL of 17 mM p-amino-benzene sulfonic acid and 2 mL of 7 mM α-naphthylamine were added, and the mixture was allowed to react for 20 min at 25 °C. The absorbance was measured at 530 nm, with a standard curve generated using NaNO_2_ [22].

The hydrogen peroxide (H_2_O_2_) content was measured with the method of Alexieva et al. [23] with slight modifications. Briefly, 0.2 g of grape fruit tissue was homogenized in 1 mL of 0.5% TCA on ice and centrifuged at 12,000× *g* for 15 min at 4 °C. A 0.5 mL aliquot of the supernatant was mixed with 0.5 mL of 100 mM potassium phosphate buffer (pH 7.0) and 0.5 mL of 1 M KI and then incubated in the dark for 1 h. The absorbance was measured at 390 nm, and the H_2_O_2_ concentration was determined on the basis of a standard curve generated using known concentrations of H_2_O_2_.

### 2.4. Enzyme Assays

Lipoxygenase (LOX) activity was measured using the method of Rao et al. [24], with one unit defined as the amount of enzyme that causes a 0.01 increase in absorbance per minute at 234 nm when linoleic acid is used at 25 °C. Superoxide dismutase (SOD) activity was assessed with a GSH assay kit protocol (Jiancheng Bioengineering Institute, Nanjing, China). Ascorbate peroxidase (APX) activity was determined with the method of Nakano and Asada [25], using a reaction mixture of enzyme extract, phosphate buffer (pH 7.0), ascorbic acid, and H_2_O_2_, and the absorbance was measured at 290 nm.

### 2.5. Determination of the Respiration Rate and Ethylene Production

The fruit respiration rate and ethylene production were determined by quantifying the CO_2_ and ethylene (C_2_H_4_) production rates using a gas chromatograph (SP9890, Lunan Ruihong Instrument Co., Tengzhou, China) with nitrogen (55 mL min^−1^) and hydrogen (25 mL min^−1^) as carrier and fuel gases, respectively [26]. The instrument parameters were maintained at 80 °C (inlet), 100 °C (column), 160 °C (detector), and 360 °C (converter furnace). For each treatment, three biological replicates were sealed in 2.25 L containers at 20 °C for 1 h, followed by headspace gas sampling (1 mL) with syringe injection. The results, expressed as mg kg^−1^ h^−1^ (CO_2_) and μL kg^−1^ h^−1^ (C_2_H_4_), were obtained from triplicate analytical measurements per sample.

### 2.6. RNA Extraction, Library Construction, Sequencing, and Data Processing

The fruit samples of ‘Shine Muscat’ grapes were subjected to cryogenic grinding in liquid nitrogen for RNA extraction. Total RNA was isolated from each sample using a Quick RNA isolation kit (Bioteke Corporation, Wuxi, China). RNA quality was assessed using 1% agarose gel electrophoresis to evaluate contamination and degradation. The RNA purity was determined using a NanoPhotometer^®^ spectrophotometer (IMPLEN, Westlake Village, CA, USA), whereas the RNA concentration was quantified with a Qubit 2.0 fluorometer (Life Technologies, Carlsbad, CA, USA). RNA integrity was assessed using an Agilent 2100 Bioanalyzer (Agilent Technologies, Santa Clara, CA, USA) [27]. Subsequently, the total RNA samples were subsequently sent to Beijing Novogene Bioinformatics Technology Co., Ltd. (Beijing, China) for cDNA library construction and de novo transcriptome sequencing on the Illumina NovaSeq X Plus platform. The raw sequencing data were deposited in the Sequence Read Archive (SRA) database under accession numbers SRX26798407-SRX26798415. Clean data were obtained by removing reads containing adapters, low-quality reads, and reads containing poly-N sequences [28]. High-quality clean reads were then utilized for subsequent bioinformatics analysis. Differential gene expression analysis was conducted based on FPKM values, with statistical analysis performed using the DESeq2 software (version 1.20.0). Genes with *p* < 0.05 and |log2FoldChange| > 1 were classified as differentially expressed genes (DEGs). To further explore the functional implications of the DEGs, Gene Ontology (GO) functional enrichment analysis (*p* < 0.05) and Kyoto Encyclopedia of Genes and Genomes (KEGG) pathway enrichment analysis (*p* < 0.01) were conducted following the methodologies outlined by Young et al. [29] and Mao et al. [30].

### 2.7. Data Statistics and Analysis

The data illustrated in all graphical representations were analyzed using one-way analysis of variance (ANOVA) conducted with SPSS statistical software (SPSS for MAC, version 21), with statistical significance defined at *p* < 0.05. The error bars depicted in the figures correspond to the standard error of the mean (±SE). All graphical data visualizations were constructed utilizing GraphPad Prism 8 software (version 9.0.0) [27].

## 3. Results

### 3.1. Effects of S. albulus Ah11601 Fermentation Broth (SFB) Treatment on the Hardness and Vitamin C (Vc) Content of ‘Shine Muscat’ Grapes

**Figure 1:**
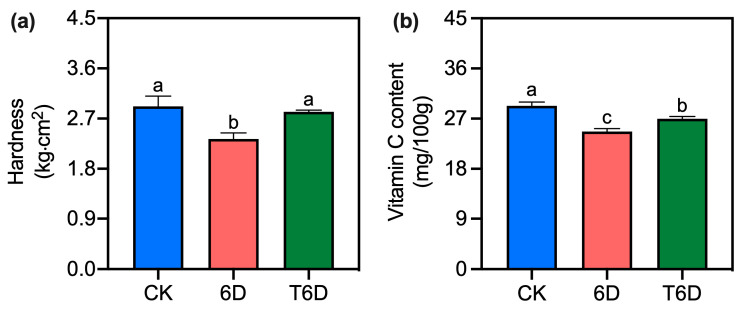
SFB effects on firmness and vitamin C content in ‘Shine Muscat’ grapes. (**a**) Hardness changes under different storage treatments. (**b**) Vitamin C content changes under different storage treatments. CK represents the untreated control, 6D denotes ‘Shine Muscat’ grapes stored at room temperature for 6 days, and T6D refers to grapes treated with SFB and stored under the same conditions for 6 days. Data are presented as mean values ± standard error (SE). Distinct lowercase letters denote statistically significant differences (*p* < 0.05) among treatments.

We initially examined the impact of Ah11601 fermentation broth (SFB) treatment on the hardness and Vc content of ‘Shine Muscat’ grapes. After 6 days of storage at room temperature (6D), grape hardness was significantly lower than that in the control group (CK), whereas no significant change in hardness was observed under the SFB treatment (T6D) (Figure 1). Although the Vc content decreased significantly under both the 6D and T6D conditions, the T6D group presented a significantly greater level of Vc relative to the 6D group.

### 3.2. Effects of SFB Treatment on the Lipooxygenase Activity and Malondialdehyde Content in ‘Shine Muscat’ Grapes

**Figure 2:**
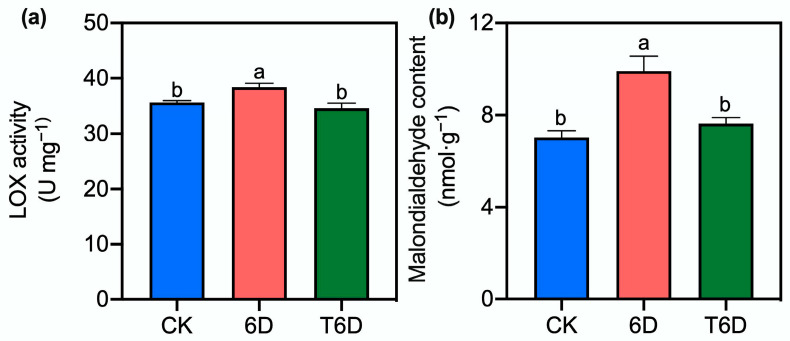
SFB effects on lipoxygenase (LOX) activity and malondialdehyde (MDA) content in ‘Shine Muscat grapes’. (**a**) LOX activity changes under different storage treatments. (**b**) MDA content variations under different storage treatments. CK represents the untreated control, 6D denotes ‘Shine Muscat’ grapes stored at room temperature for 6 days, and T6D refers to grapes treated with SFB and stored under the same conditions for 6 days. Data are presented as mean values ± standard error (SE). Distinct lowercase letters denote statistically significant differences (*p* < 0.05) among treatments.

Compared with that in CK grapes, the lipoxygenase (LOX) enzyme activity in ‘Shine Muscat’ grapes was significantly elevated after 6 days of treatment (Figure 2). In contrast, no significant change in LOX activity was observed with T6D treatment. Similarly, the malondialdehyde (MDA) content increased by 40.8% relative to that of the CK, whereas the T6D treatment effectively maintained MDA levels at normal levels.

### 3.3. Effects of SFB Treatment on the Generation Rate of Superoxide Radicals and Content of Hydrogen Peroxide in ‘Shine Muscat’ Grapes

**Figure 3:**
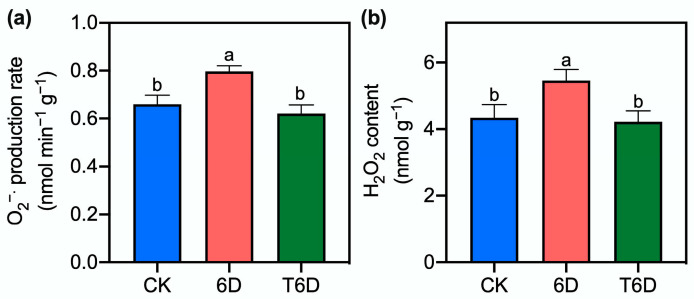
SFB effects on the generation rate of superoxide radical (O_2_^−^) and hydrogen peroxide (H_2_O_2_) content in ‘Shine Muscat’ grapes. (**a**) Differential patterns of O_2_^−^ production across treatment groups (**b**) H_2_O_2_ content variations across treatment groups. CK represents the untreated control, 6D denotes ‘Shine Muscat’ grapes stored at room temperature for 6 days, and T6D refers to grapes treated with SFB and stored under the same conditions for 6 days. Data are presented as mean values ± standard error (SE). Distinct lowercase letters denote statistically significant differences (*p* < 0.05) among treatments.

Following a 6-day period of conventional storage at room temperature, the production rate of superoxide radical (O_2_^−^) in ‘Shine Muscat’ grapes was significantly greater than that in the control group (Figure 3). In contrast, the application of T6D treatment markedly suppressed the formation of O_2_^−^. Concurrently, the hydrogen peroxide (H_2_O_2_) concentration substantially increased under the 6D condition compared with that of the control, whereas T6D intervention effectively stabilized H_2_O_2_ levels within the normative range.

### 3.4. Effects of SFB Treatment on Superoxide Dismutase and Ascorbate Peroxidase Enzyme Activities in ‘Shine Muscat’ Grapes

**Figure 4:**
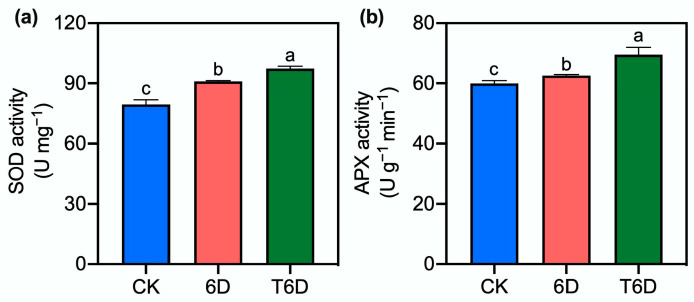
SFB effects on the activity of superoxide dismutase (SOD) and ascorbate peroxidase (APX) in ‘Shine Muscat’ grapes. (**a**) SOD activity changes under different storage treatments. (**b**) APX activity changes under different storage treatments. CK represents the untreated control, 6D denotes ‘Shine Muscat’ grapes stored at room temperature for 6 days, and T6D refers to grapes treated with SFB and stored under the same conditions for 6 days. Data are presented as mean values ± standard error (SE). Distinct lowercase letters denote statistically significant differences (*p* < 0.05) among treatments.

The superoxide dismutase (SOD) activity in ‘Shine Muscat’ grapes significantly increased after 6 days of storage at room temperature, with a more pronounced increase under the T6D treatment (Figure 4). Similarly, ascorbate peroxidase (APX) activity markedly increased under both the 6D and T6D treatments compared with the CK treatment, with T6D exhibiting a 16% increase over the CK treatment and an 11.2% increase over the 6D treatment.

### 3.5. Effects of SFB Treatment on the Respiratory Rate and Ethylene Production in ‘Shine Muscat’ Grapes

**Figure 5:**
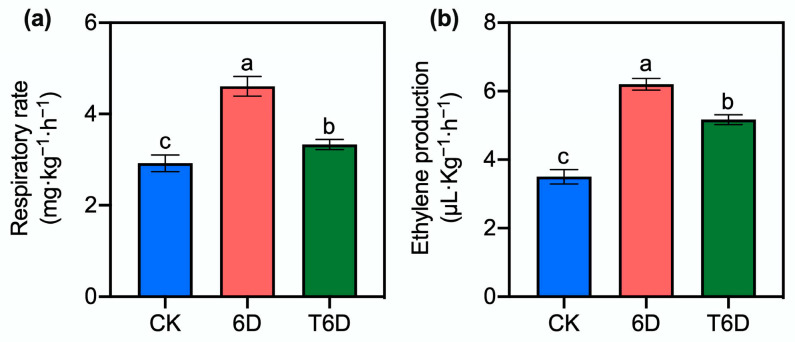
SFB effects on the respiratory rate and ethylene production in ‘Shine Muscat’ grapes. (**a**) Respiratory rate changes under different storage treatments. (**b**) Ethylene production changes under different storage treatments. CK represents the untreated control, 6D denotes ‘Shine Muscat’ grapes stored at room temperature for 6 days, and T6D refers to grapes treated with SFB and stored under the same conditions for 6 days. Data are presented as mean values ± standard error (SE). Distinct lowercase letters denote statistically significant differences (*p* < 0.05) among treatments.

The respiratory rate of ‘Shine Muscat’ grapes was significantly increased under both 6D and T6D treatments (Figure 5a). However, this parameter was significantly lower in the T6D treatment than in the 6D treatment. Similarly, the ethylene production increased markedly in both the 6D and T6D treatment groups relative to that in the CK group (Figure 5b), but significantly decreased in the T6D treatment group versus the 6D treatment group.

### 3.6. Transcriptome Data Assembly and Functional Annotation

High-throughput sequencing of the T6D, 6D, and CK samples was performed using the Illumina NovaSeq X Plus sequencing platform. The raw sequencing reads underwent rigorous preprocessing, which yielded a comprehensive dataset comprising 37,951,524 to 45,890,384 high-quality clean reads. The quality metrics Q20 and Q30 ranged from 98.08% to 98.47% and 94.55% to 95.66%, respectively, indicating of the high fidelity of the sequencing data. Moreover, the GC content was quantified to be within the range of 46.33% to 47.09%.

**Figure 6:**
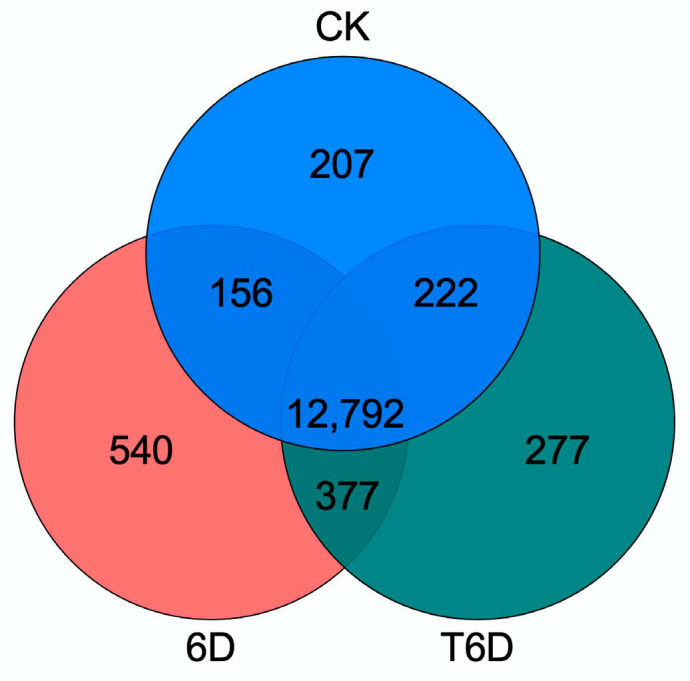
Venn diagram of transcriptome genes in ‘Shine Muscat’ grapes. CK represents the untreated control, 6D denotes ‘Shine Muscat’ grapes stored at room temperature for 6 days, and T6D refers to grapes treated with SFB and stored under the same conditions for 6 days.

**Figure 7:**
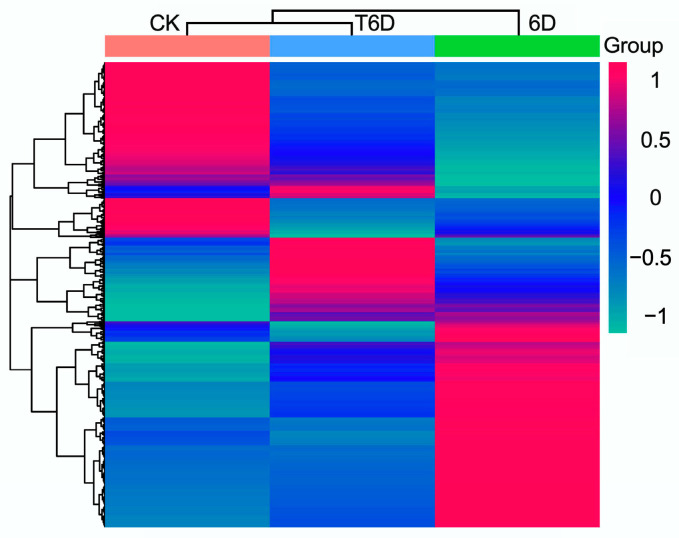
Cluster analysis of transcriptome genes in ‘Shine Muscat’ grapes. CK represents the untreated control, 6D denotes ‘Shine Muscat’ grapes stored at room temperature for 6 days, and T6D refers to grapes treated with SFB and stored under the same conditions for 6 days.

Venn diagram analysis revealed a conserved set of 12,792 genes coexpressed across CK, 6D, and T6D, whereas 207, 540, and 277 genes exhibited exclusive expression in CK, 6D, and T6D, respectively. Furthermore, pairwise comparative analysis identified 156, 222, and 377 genes coexpressed between CK and 6D, CK and T6D, and 6D and T6D, respectively (Figure 6). Subsequent transcriptomic profiling and hierarchical clustering analysis revealed that the identified genes were systematically classified into five distinct functional categories, underscoring the transcriptional complexity and regulatory divergence among the experimental groups (Figure 7).

### 3.7. Screening of Differentially Expressed Genes

**Figure 8:**
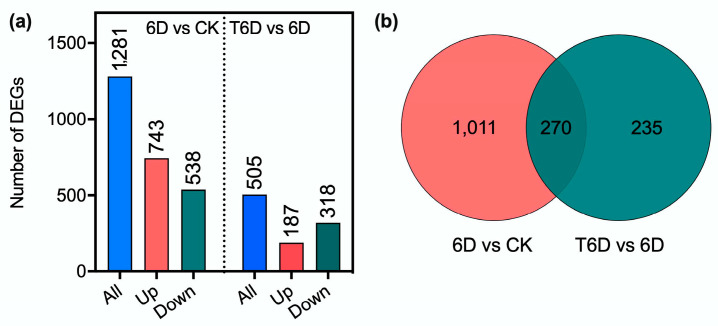
Statistical analysis of differentially expressed genes (DEGs) (**a**) and Venn diagram (**b**). CK represents the untreated control, 6D denotes ‘Shine Muscat’ grapes stored at room temperature for 6 days, and T6D refers to grapes treated with SFB and stored under the same conditions for 6 days.

Differentially expressed genes (DEGs) associated with the preservation effect of SFB on ‘Shine Muscat’ grapes were identified using the criteria of *p*_adj_ ≤ 0.05 and |log2Fold Change| ≥ 1. A total of 1281 DEGs were detected in the 6D vs. CK transcriptome, with 743 genes upregulated and 538 downregulated (Figure 8a). In the T6D vs. 6D comparison, 505 DEGs were identified, comprising 187 upregulated and 318 downregulated genes. The Venn diagram analysis indicated that 270 DEGs were shared between the 6D vs. CK and T6D vs. 6D comparisons. In addition, 1011 DEGs were uniquely found in the 6D vs. CK group, and 235 DEGs were exclusive to the T6D vs. 6D group (Figure 8b).

### 3.8. Gene Ontology and Kyoto Encyclopedia of Genes and Genomes Enrichment Analyses of DEGs

**Figure 9:**
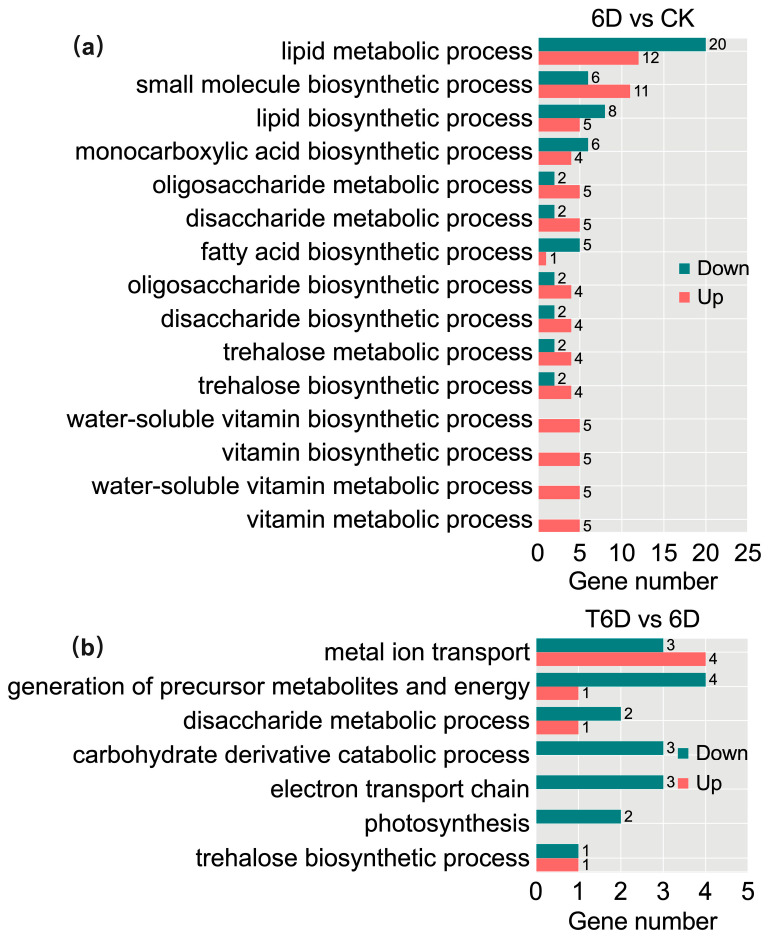
Gene Ontology (GO) enrichment analysis of DEGs. Comparative analysis between 6D and CK (**a**). Comparative analysis between T6D and 6D (**b**). The red and green colors denote significant GO enrichment terms associated with upregulated and downregulated DEGs, respectively. CK represents the untreated control, 6D denotes ‘Shine Muscat’ grapes stored at room temperature for 6 days, and T6D refers to grapes treated with SFB and stored under the same conditions for 6 days.

To investigate the functional distribution, biological processes, and associated metabolic pathways of the DEGs, Gene Ontology (GO) and Kyoto Encyclopedia of Genes and Genomes (KEGG) enrichment analyses were conducted. The results from the GO enrichment analysis revealed that, in the comparison of 6D and CK, DEGs were significantly enriched in lipid metabolism, including lipid metabolic process (GO:0006629), lipid biosynthetic process (GO:0008610), and fatty acid biosynthetic process (GO:0006633); carbohydrate metabolism, such as trehalose biosynthetic process (GO:0005992), trehalose metabolic process (GO:0005991), disaccharide biosynthetic process (GO:0046351), oligosaccharide biosynthetic process (GO:0009312), disaccharide metabolic process (GO:0005984), and oligosaccharide metabolic process (GO:0009311); and vitamin metabolism, comprising vitamin metabolic process (GO:0006766), water-soluble vitamin metabolic process (GO:0006767), vitamin biosynthetic process (GO:0009110), and water-soluble vitamin biosynthetic process (GO:0042364) (Figure 9a). In contrast, in the T6D vs. 6D comparison, DEGs were predominantly enriched in metal ion transport (GO:0030001), generation of precursor metabolites and energy (GO:0006091), carbohydrate metabolism and related pathways such as trehalose biosynthetic process (GO:0005992), disaccharide metabolic process (GO:0005984), and carbohydrate derivative catabolic process (GO:1901136), as well as photosynthesis-related pathways, including photosynthesis, light reaction (GO:0019684), and the electron transport chain (GO:0022900) (Figure 9b).

**Figure 10:**
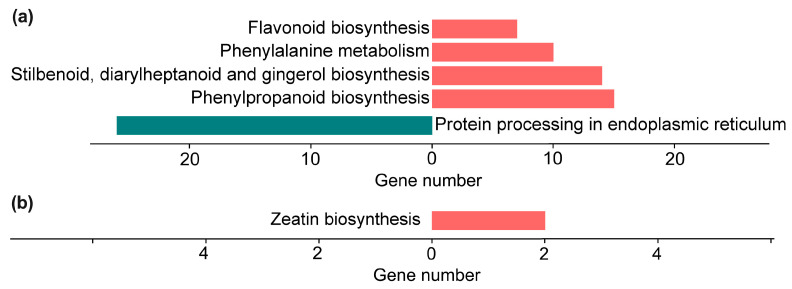
Kyoto Encyclopedia of Genes and Genomes (KEGG) analysis of DEGs. Comparative analysis between 6D and CK (**a**). Comparative analysis between T6D and 6D (**b**). The red and green colors denote significant KEGG enrichment pathways associated with upregulated and downregulated DEGs, respectively. CK represents the untreated control, 6D denotes ‘Shine Muscat’ grapes stored at room temperature for 6 days, and T6D refers to grapes treated with SFB and stored under the same conditions for 6 days.

**Figure 11:**
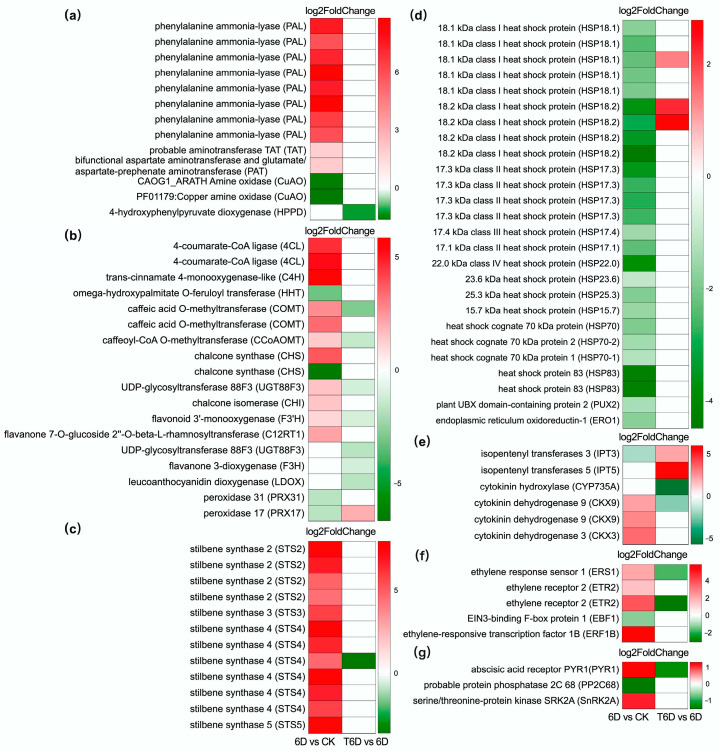
DEGs related to phenylalanine metabolism (**a**); phenylpropanoid metabolism (**b**); stilbene, diarylheptanoid, and gingerol biosynthesis (**c**); zeatin biosynthesis (**d**); protein processing in the endoplasmic reticulum (**e**); and ethylene (**f**) and abscisic acid (**g**) signaling pathways. The upregulated and downregulated DEGs are represented in red and green, respectively, with the color bar reflecting the relative intensity of gene expression.

The KEGG enrichment analysis revealed that, in the 6D vs. CK comparison, the upregulated DEGs were significantly enriched in key metabolic pathways, including phenylpropanoid biosynthesis (vvi00940), flavonoid biosynthesis (vvi00941), phenylalanine metabolism (vvi00360), and stilbenoid, diarylheptanoid, and gingerol biosynthesis (vvi00945) (Figure 10a), with each gene showing differential expression at the transcriptional level (Figure 11a–c). In the same 6D vs. CK comparison, the downregulated DEGs were significantly enriched in protein processing in endoplasmic reticulum (vvi04141) (Figure 10a and Figure 11d). In contrast, following treatment with SBF (T6D vs. 6D), the upregulated DEGs were predominantly enriched in zeatin biosynthesis (vvi00908) (Figure 10b and Figure 11e). Furthermore, significant enrichment of DEGs associated with the ethylene and abscisic acid signaling pathways was also observed (Figure 11f,g).

### 3.9. Construction and Module Analysis of the Protein–Protein Interaction Networks

**Figure 12:**
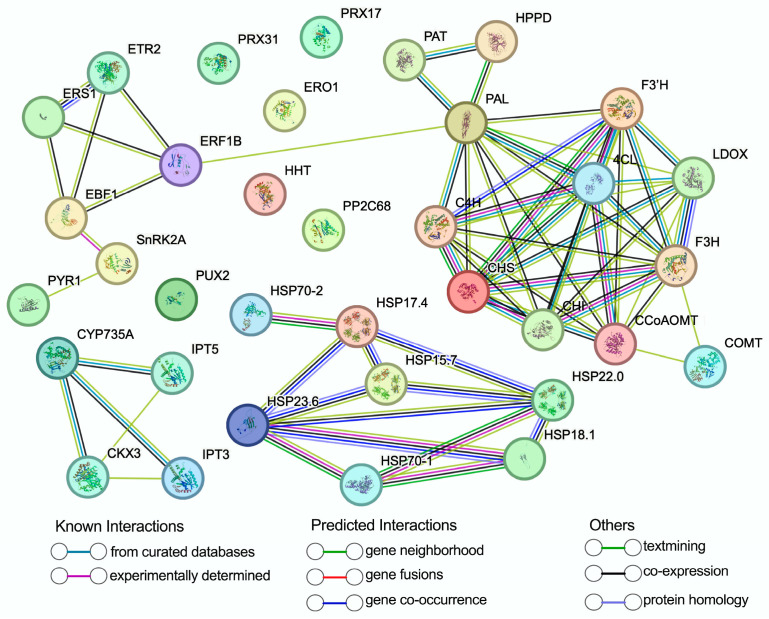
The protein–protein interaction (PPI) network of DEGs based on the results of functional enrichment analysis.

**Figure 13:**
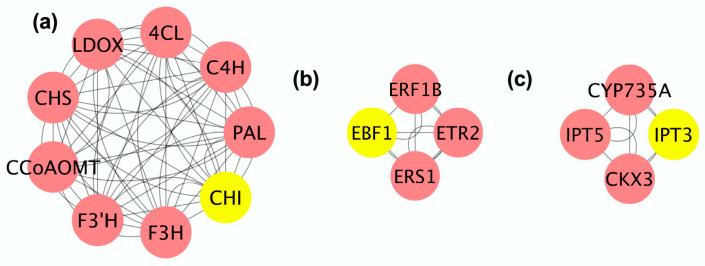
Clustering analysis of PPI network of identified DEGs. (**a**–**c**) Protein compositions of clusters 1–3, where nodes represent individual proteins, edges denote interactions, and predicted seed nodes are highlighted with yellow circles.

Utilizing the STRING online database and Cytoscape software (version 3.7.1), a protein–protein interaction (PPI) network was constructed using the DEGs to elucidate potential functional modules modulated by SFB in the postharvest preservation of ‘Shine Muscat’ grapes. The PPI network analysis revealed a total of 35 nodes and 66 interaction edges under the 6D and T6D treatment conditions (Figure 12 and Table S1). Moreover, three distinct functional modules were delineated utilizing the MCODE plugin (Figure 13 and Table S2). Cluster 1, comprising nine nodes, was characterized by caffeoyl-CoA O-methyltransferase (CCoAOMT) as the seed node and was predominantly implicated in the phenylpropanoid biosynthetic pathway (Figure 13 and Table S2). Clusters 2 and 3, each encompassing four nodes, were anchored by EIN3-binding F-box protein 1 (EBF1) and isopentenyl transferases 3 (IPT3) as their respective seed nodes. These clusters were significantly associated with the ethylene signaling and zeatin biosynthesis, highlighting their critical roles in these metabolic and regulatory processes.

## 4. Discussion

During postharvest storage, grape berries undergo progressive senescence due to normal metabolic activities (e.g., respiration and transpiration) and pathogenic infections. This process leads to significant alterations in fruit color, texture, flavor profiles, and taste characteristics. Concurrently, visible quality deterioration manifests as water loss, berry shattering, stem browning, rot development, and enzymatic browning, all of which directly compromise visual quality and market value [31]. Given these challenges, biologically based preservation strategies have gained attention for their potential to mitigate postharvest losses. Growing evidence shows that actinobacterial metabolites effectively inhibit postharvest pathogen growth in fruits and vegetables [10,11,13,14]. On the basis of these findings, this study investigated the novel investigation into the preservation efficacy of *S. albulus* Ah11601 fermentation broth (SFB) application on ‘Shine Muscat’ grapes.

To understand the mechanisms underlying the preservation effects of SFB, we examined its impact on cell wall integrity, which is a critical factor in fruit softening. Our results revealed that 6D treatment significantly reduced the hardness of ‘Shine Muscat’ grapes (Figure 1), whereas SFB application effectively counteracted this softening, demonstrating its preservation potential. In addition to physical texture, nutritional quality is another key indicator of postharvest deterioration. The level of vitamin C (Vc), a key antioxidant in ‘Shine Muscat’ grapes, reflects nutritional quality and storability. In this study, compared with the CK treatment, both the 6D and T6D treatments significantly reduced the Vc content (Figure 1). The decrease in Vc content might stem from oxidative degradation during storage and room temperature-induced AsA–GSH activation, along with water loss and ROS scavenging. Notably, T6D treatment significantly reduced Vc degradation compared with that of 6D, further demonstrating the antioxidative protection of the SFB coating. Oxidative stress and membrane damage are hallmarks of postharvest senescence. When plant tissues are exposed to environmental stress, LOX is activated and facilitates the peroxidation of membrane lipids, leading to the degradation of cell membranes and the subsequent accumulation of MDA, a key metabolic byproduct. In this study, compared with the CK treatment, the 6D treatment significantly increased the LOX activity and MDA content in ‘Shine Muscat’ grapes (Figure 2), indicating severe membrane lipid peroxidation damage to the cellular membrane system. In contrast, the T6D treatment markedly suppressed both LOX activity and MDA accumulation, confirming that the SFB coating effectively reduces the extent of lipid peroxidation and alleviates oxidative stress-induced structural damage to the fruit membrane, thereby maintaining postharvest fruit quality. The role of ROS in postharvest deterioration further underscores the importance of antioxidant defenses. Under normal conditions, the formation and elimination of ROS in plants are balanced in a dynamic equilibrium [32]; however, stress conditions perturb this balance. Our results revealed that the 6D treatment significantly elevated both H_2_O_2_ and O_2_^−^ levels (Figure 3). These findings suggest that 6 days of room temperature storage induces an increase in ROS, leading to impaired membrane integrity. Notably, the T6D treatment significantly inhibited H_2_O_2_ accumulation and O_2_^−^ production (Figure 3). The underlying mechanisms may involve physical barrier effects limiting oxygen permeability, the activation of endogenous antioxidant enzymes, and the maintenance of membrane lipid molecular ordering. These synergistic effects collectively retard the progression of membrane oxidative damage, consequently inhibiting postharvest quality deterioration. To further elucidate the antioxidant response, we analyzed enzymatic activities. Consistent with these findings, both SOD and APX activities were markedly increased under the 6D and T6D treatments, with T6D resulting in significantly greater enzymatic activity than did 6D (Figure 4). The observed fluctuations in SOD activity provide critical insights into the oxidative stress response capacity of plants, where SOD catalyzes the dismutation of O_2_^−^ to H_2_O_2_ as the primary defense against free radical damage [33]. APX, a core component of the AsA–GSH cycle, plays a vital role in H_2_O_2_ detoxification [32]. Therefore, we conclude that the SBF coating significantly enhances SOD and APX activities in ‘Shine Muscat’ grapes, facilitating ROS scavenging and subsequently reducing lipid peroxidation levels, oxidative stress-induced membrane damage, and quality deterioration during storage. Through these coordinated mechanisms regulating redox homeostasis, SBF treatment has demonstrated remarkable efficacy in preserving postharvest fruit quality. The present findings align with previous studies demonstrating that treatment with *B. velezensis* HY19 fermentation broth (BFB) in citrus [13], as well as the application of *Bacillus* XT1 CECT 8661-derived lipopeptides in tomatoes and grapes [34]. Specifically, both BFB and lipopeptide treatments significantly increased the antioxidant capacity, induced systemic resistance against phytopathogens, and improved the postharvest shelf life. In contrast, conflicting evidence exists regarding defense enzyme modulation. For instance, *Trichoderma harzianum* PBT-23-primed mustard seeds challenged with *Alternaria brassicae* after sowing 60 days presented upregulated CAT and SOD activities, whereas APX and POD levels remained unaltered [35]. These observations underscore the intricate and species-specific regulatory mechanisms mediated by microbial-derived bioactive compounds in postharvest systems, highlighting the need for tailored biocontrol strategies across different crops. Collectively, physiological and biochemical evidence demonstrated that the 6D treatment adversely affected the postharvest quality maintenance of ‘Shine Muscat’ grapes, whereas the SFB treatment had significant preservation benefits. To bridge these physiological observations with molecular regulation, we conducted transcriptomic analysis. Transcriptome profiling revealed that key metabolic pathways including zeatin biosynthesis, ethylene and abscisic acid signaling pathways, secondary metabolite biosynthesis, and protein processing in endoplasmic reticulum play crucial roles in the SFB-mediated preservation of ‘Shine Muscat’ grapes.

Building upon these transcriptomic findings, we first focused on hormone-regulated pathways. Plant hormones are a class of trace organic compounds that are produced during normal plant metabolism and play pivotal roles in regulating growth, development, and responses to environmental stress. These hormones include auxin (AUX), cytokinin (CTK), gibberellin (GA), ethylene (ETH), abscisic acid (ABA), and others. Specifically, we identified DEGs linked to the CTK biosynthesis as well as ETH and ABA signaling pathways, which collectively participate in the SFB-mediated preservation mechanism of ‘Shine Muscat’ grapes. Zeatin, a key type of CTK, is essential for regulating both cell growth and differentiation. In this study, six genes associated with zeatin biosynthesis were identified, comprising two isopentenyl transferase genes (IPTs), one CTK hydroxylase gene (CYP735A), and three CTK dehydrogenase 9 genes (CKX9) (Figure 11). Previous studies have indicated that isoprenoid derivatives, including trans-zeatin (tZ), isopentenyl adenine (iP), cis-zeatin (cZ), and dihydrozeatin (DZ), as well as their nucleoside, nucleotide, and glucoside forms, are the predominant forms of CTK in plants [19,20]. The efficacy of these CTK derivatives, depending on their structural forms, can vary considerably across different plant species, tissues, or developmental stages. Additionally, these compounds may undergo interconversion under specific conditions, which helps to regulate and maintain the activity levels of free CTK [21]. The biosynthesis of CTK de novo, particularly through the use of isopentenyl side chains as precursors, represents the primary pathway for the production of endogenous CTK. The enzyme IPT, which is rate limiting in CTK biosynthesis, catalyzes the conversion of adenosine triphosphate (ATP), adenosine diphosphate (ADP), or adenosine monophosphate (AMP) with dimethylallyl diphosphate (DMAPP) to form isopentenyl adenosine phosphates (iPRTP/iPRDP/iPRMP), which subsequently leads to the production of isopentenyl adenosine (iPR). CYP735A then converts these isopentenyl adenosine phosphates into trans-zeatin riboside (tZR), which is ultimately synthesized into tZ. Additionally, the LONELY GUY (LOG) gene encodes a riboside hydrolase, further converting iPR and tZR into biologically active, free radical forms of iP and tZ. Furthermore, CTK degradation in plants is predominantly catalyzed by CTK oxidase/dehydrogenase (CKX), an enzyme that cleaves the unsaturated bond in CTK, yielding adenine and isopentenal, leading to irreversible degradation [22]. In this study, under 6D treatment, the downregulation of IPT3 and the upregulation of CKX3 and CKX9 suggested a reduction in CTK levels. However, following the application of SFB, the expression of genes related to zeatin biosynthesis was modulated, with IPT3 and IPT5 being upregulated and CYP735A2 and CKX9 being downregulated. This modulation may help maintain elevated levels of iP, which could play a critical role in the preservation of ‘Shine Muscat’ grapes.

In addition to CTK metabolism, ETH and ABA signaling also presented pronounced changes (Figure 11). ETH, a naturally occurring plant hormone, has been shown to accelerate postharvest senescence and deterioration of fresh produce, significantly reducing shelf life. The action of ethylene necessitates the activation of specific signaling pathways [36,37]. Following the 6D treatment, five DEGs were identified, including one gene encoding ETH response sensor 1 (ERS1), two genes encoding ETH receptor 2 (ETR2), one gene encoding EIN3-binding F-box protein 1 (EBF1), and one gene encoding ETH-responsive transcription factor 1B (ERF1B). ERS1 and ETR2 are key ETH receptors, and upon ETH binding, these receptors inactivate the constitutive triple response 1 (CTR1) complex, leading to the activation of the downstream signaling component ETH-insensitive 2 (EIN2). EIN2 subsequently transmits the ETH signal to the nucleus, where it activates EIN3 and triggers the transcriptional activation of downstream target genes such as ERF1. Furthermore, the protein level of EIN3 is predominantly regulated through the ubiquitination pathway. During the ubiquitin-mediated degradation of EIN3, two F-box proteins, EBF1 and EBF2, play crucial roles in its recognition. In this study, the upregulation of ERS1, ETR2, and ERF1B, coupled with the downregulation of EBF1, suggests the activation of the ETH signaling pathway following 6D treatment, thus accelerating the ripening and senescence of ‘Shine Muscat’ grapes. These results are consistent with those of a previous study, which demonstrated that MaERF012 modulates banana ripening by promoting fruit yellowing and softening [38]. Conversely, the downregulation of ERS1 and ETR2 following T6D treatment indicates that SFB may contribute to the preservation of ‘Shine Muscat’ grapes by inhibiting the ethylene signaling pathway. Our physiological data further confirmed these findings, with both ethylene formation and the respiration rate (Figure 5) being markedly lower in T6D than in 6D. A previous study supported our finding that 1-methylcyclopropene (an ethylene inhibitor) maintained sand pear firmness and quality by reducing respiration, suppressing ETH/ABA biosynthesis, and enhancing antioxidant activity during room-temperature storage [39]. These results suggest that SFB modulates fruit ripening progression by suppressing ethylene biosynthesis and the respiration rate, delaying associated physiological and biochemical responses (including slower declines in fruit firmness and Vc content, along with reduced ROS accumulation).

ABA is another critical hormone that regulates fruit ripening and plays a predominant role in the maturation of nonclimacteric fruits. Its synthesis and signaling pathways govern the regulation of ripening processes. Research has demonstrated that a rapid increase in endogenous ABA levels can promote fruit ripening, and that exogenous ABA application can significantly accelerate the maturation of nonclimacteric fruits [40,41]. In the present study, following the 6D treatment, three DEGs involved in the ABA signaling pathway were identified (Figure 11): ABA receptor PYR1 (PYR1), probable protein phosphatase 2C 6 (PP2C06), and serine/threonine-protein kinase SRK2A (SnRK2A). The overexpression of the tomato ABA receptor gene *SlPYL9* has been shown to effectively promotes the expression of downstream response factors such as *SlPP2C1/2/9*, *SnRK2.8*, and *SlABF2*, thereby accelerating fruit ripening [42]. Conversely, the tomato *SlPP2C1* gene peaks in expression during the precoloring phase, and silencing this gene leads to premature fruit ripening and accelerated color change [43]. In addition, preliminary RNA-Seq data suggested that ETH and ABA biosynthesis and signaling genes, including *ACS2/4*, *ACO1/4/5*, *EBF*, *NCED*, *ABA8ox1*, and *PYR1/PYL4*, may serve as key regulators in coordinating postharvest fruit senescence in cherry tomatoes [44]. Similarly, on the basis of these findings, we propose that under the 6D treatment, ABA binds to PYR1, which inhibits the enzyme activity of PP2C06, reducing the stability of the PP2C–SnRK2A complex. This results in the dissociation of SnRK2 from PP2C, thereby activating SnRK2A phosphorylation and regulating the ripening process of ‘Shine Muscat’ grapes. In contrast, T6D treatment appears to inhibit the ABA signaling pathway by downregulating the transcription of SnRK2A, contributing to the preservation of ‘Shine Muscat’ grapes.

Furthermore, RNA-seq analysis demonstrated the functional engagement of secondary metabolite biosynthesis-associated DEGs in SFB-induced quality maintenance of ‘Shine Muscat’ grapes. Secondary metabolites, including phenylpropanoids and their derivatives, terpenoids, and alkaloids, are widely distributed in plants. These compounds play pivotal roles as bioactive molecules or signaling agents, regulating plant growth, development, and defense mechanisms against microbial pathogens, viral infections, herbivory, and interspecies competition [45,46]. In the present study, ‘Shine Muscat’ grapes subjected to a six-day room temperature storage period presented marked upregulation of gene expression within secondary metabolic pathways associated with defense responses. These pathways include flavonoid biosynthesis and phenylpropanoid biosynthesis as well as the biosynthesis of stilbenes, diarylheptanoids, and gingerol (Figure 11). These findings demonstrate that 6D treatment elicited the production of a substantial array of defense-related secondary metabolites in ‘Shine Muscat’ grapes, which are critically implicated in determining the qualitative attributes of the fruit.

The phenylpropanoid metabolic pathway represents one of the pivotal pathways in the biosynthesis of plant secondary metabolites and encompasses a series of critical biochemical processes. This pathway is focused primarily on the metabolism of phenylalanine and extends to downstream branches, facilitating the synthesis of diverse secondary metabolites, including flavonoids, lignin, and chlorogenic acid [47]. In this study, a total of 12 DEGs associated with the phenylalanine metabolic pathway were identified following the 6D treatment, with 10 genes exhibiting upregulation and two genes demonstrating downregulation (Figure 11). This observation suggests that the upregulation of the phenylalanine metabolic pathway may contribute to phenylpropanoid metabolism by providing more phenylalanine substrates. Research indicates that the phenylpropanoid metabolic pathway involves the participation of multiple key enzymes. Phenylalanine undergoes deamination catalyzed by phenylalanine ammonia-lyase (PAL) to yield trans-cinnamic acid, which is subsequently hydroxylated by cinnamate-4-hydroxylase (C4H) to produce trans-4-coumaric acid. This intermediate is further metabolized by key enzymes, including coumarate 3-hydroxylase (C3H), 4-coumarate-CoA ligase (4CL), hydroxycinnamoyl-CoA/quinate hydroxycinnamoyltransferase (HCT), and chalcone synthase (CHS), culminating in the biosynthesis of lignin, chlorogenic acid, and flavonoids. In this study, following the 6D treatment, eight genes encoding PAL, two genes encoding 4CL, one gene encoding C4H, and two genes encoding CHS were identified (Figure 11). The majority of these genes were upregulated after 6D treatment, suggesting that flavonoid metabolism and associated downstream pathways may play roles in the ripening and senescence mechanisms of ‘Shine Muscat’ grapes during room temperature storage.

Indeed, transcriptomic profiling revealed a total of eight DEGs associated with the flavonoid metabolic pathway, with six genes upregulated and two genes downregulated. Recent studies have underscored the importance of secondary metabolites derived from the flavonoid pathway in plant growth, development, pest resistance, and cell wall composition [48,49]. Furthermore, flavonoids exhibit potent antimicrobial and antioxidant activities under environmental stress, directly or indirectly scavenging free radicals and bolstering plant resistance to pathogens, a phenomenon closely linked to the senescence of fruits and vegetables [48,49,50]. This study further demonstrated that after 6 days of room temperature storage, the levels of MDA and H_2_O_2_ and the generation rate of O_2_^−^ in ‘Shine Muscat’ grapes were significantly elevated (Figure 2 and Figure 3). Therefore, we hypothesize that the 6D treatment may increase the antimicrobial and antioxidant capacity of ‘Shine Muscat’ grapes by upregulating genes associated with the flavonoid metabolic pathway. Moreover, following treatment with SFB, all six DEGs identified in the flavonoid pathway were downregulated, concomitant with reduced levels of MDA and H_2_O_2_ and a decreased O_2_^−^ generation rate. These findings suggested that the T6D treatment may mitigate oxidative stress, thereby delaying senescence and extending the shelf life of ‘Shine Muscat’ grapes.

Additionally, the pathway of stilbene, diarylheptanoid, and gingerol biosynthesis was upregulated under the 6D treatment (Figure 11). Stilbenoids, also referred to as stilbenes, constitute a class of phytoalexins characterized by a stilbene core, belonging to the phenylpropanoid family, and are synthesized in response to viral, pest, or abiotic stress challenges. Studies have demonstrated that stilbene synthase (STS) is a key enzyme in the biosynthesis of resveratrol and stilbenes. Under normal physiological conditions, grapevines contain minimal levels of stilbenes; however, their content is markedly elevated under biotic and abiotic stresses because of the induction of genes encoding PAL and STS [51]. Therefore, we postulate that ‘Shine Muscat’ grapes may enhance stress resistance and maintain quality under 6D treatment by upregulating genes associated with the pathway of stilbene, diarylheptanoid, and gingerol biosynthesis, particularly *STS* genes.

This study also identified peroxidases (PRXs) associated with lignin metabolism as playing a significant role in ‘Shine Muscat’ grapes. Previous research has shown that the loss of *AtPRX2* and *AtPRX25* in *Arabidopsis* results in a substantial reduction in total lignin content, while mutations in *AtPRX2*, *AtPRX25*, and *AtPRX71* can alter lignin structure [52]. Similarly, in poplar, the expression level of the peroxidase gene *CWPO-C* is positively correlated with lignin content, and suppression of this gene reduces lignin content by approximately 45% [53]. In this study, under the 6D treatment, the expression of PRX17 and PRX31 was downregulated, whereas PRX17 transcription was significantly upregulated, indicating that the 6D treatment may inhibit lignin synthesis, potentially explaining the observed reduction in the hardness of ‘Shine Muscat’ grapes compared with the CK and T6D-treated groups.

In addition, transcriptomic profiling further revealed the critical involvement of endoplasmic reticulum (ER) protein processing-associated DEGs in the preservation mechanism of ‘Shine Muscat’ grapes mediated by SFB. The ER serves as the principal intracellular organelle responsible for protein processing, wherein only correctly folded proteins are competent to execute designated biological functions. Protein synthesis is initiated by free ribosomes within the cytosol, with a subset of proteins subsequently translocated to the ER membrane, where elongation continues and synthesis is completed [54]. In the present study, 28 DEGs associated with the ER protein processing pathway were identified following 6D and T6D treatments (Figure 11). Research has shown that nascent polypeptides are transported to the ER for posttranslational modifications and proper folding, a process mediated by ER-associated chaperones [55,56], including heat shock proteins (HSPs) and immunoglobulin heavy chain-binding protein (BiP). The overexpression of *BiP* in tobacco [57] and soybean [58] has been demonstrated to enhance drought tolerance in transgenic plants by alleviating endogenous oxidative stress. Similarly, overexpression of pepper *CaBiP1* in *Arabidopsis* resulted in diminished ROS accumulation, improved water retention capacity, activation of the unfolded protein response (UPR) pathway, and upregulation of stress-responsive genes, thereby augmenting tolerance to diverse environmental stressors such as heat, osmotic stress, salinity, and drought [59]. Furthermore, the overexpression of the *Lycium chinense LcBiP* gene in tobacco enhanced plant tolerance to the heavy metal cadmium [60]. HSPs, including HSP100, HSP90, HSP70, HSP60, and small heat shock proteins (sHSPs), are categorized into distinct families based on molecular weight and sequence homology. The latter, an evolutionarily conserved and heterogeneous protein family, is ubiquitously expressed in plants. Under standard growth conditions, sHSPs are generally undetectable in plant tissues; however, their expression is markedly induced under stress conditions or during specific developmental stages, such as high temperatures, drought, heavy metal stress, seed germination, pollen development, and fruit maturation. This implies that sHSPs function as molecular chaperones and are involved in modulating plant stress responses as well as growth, development, and senescence [61,62]. For instance, overexpression of the wheat chloroplast gene *TaHSP26* in *Arabidopsis* confers enhanced thermotolerance and seed vigor [63], while overexpression of *HaHSFA9* in sunflower (*Helianthus annuus*) increases the accumulation of CI- and CII-class sHSPs in transgenic tobacco seeds, thereby improving seed viability and extending longevity [64]. Consistent with these findings, our results demonstrated that treatment with SFB significantly upregulated the expression levels of the *BiP*, *HSP18.1*, and *HSP18.2* genes in ‘Shine Muscat’ grapes (Figure 11). These findings suggest that *BiP*, *HSP18.1*, and *HSP18.2* play critical roles in the stress response during 6 days of room temperature storage, likely contributing to the regulation of quality preservation. Conversely, following the 6D treatment, a total of 26 DEGs associated with the ER protein processing pathway were identified, all of which were downregulated. These included one gene encoding ER oxidoreductin-1 (ERO1), one gene encoding UBX domain-containing protein 2 (UbX2), three genes encoding HSP70, two genes encoding HSP90, and 19 genes encoding sHSPs. Notably, ERO1 is a pivotal oxidizing enzyme involved in protein folding. In yeast, ERO1-deficient mutants are unable to form disulfide bonds in the ER, leading to the accumulation of misfolded, reduced proteins [65,66]. ER-associated degradation (ERAD) is a specialized ubiquitin-proteasome pathway within the ER that plays a crucial role in the clearance of misfolded proteins and the mitigation of ER stress. The UBX protein family is integral to the ubiquitination process [67]. In this study, the downregulation of *ERO1*, *UBX2*, *HSP70*, *HSP90*, and *sHSPs* following the 6D treatment suggested that the ERAD pathway was suppressed during the 6-day room temperature storage of ‘Shine Muscat’ grapes. This suppression may lead to the accumulation of misfolded or improperly modified proteins within the ER, thereby exacerbating oxidative stress and potentially compromising the retention of grape fruit quality and stress resistance.

## 5. Conclusions

In summary, our findings confirm our hypothesis that SFB treatment provides significant preservation benefits for ‘Shine Muscat’ grapes. This effect is mediated through maintaining cellular redox homeostasis, modulating cytokinin and ethylene biosynthesis, and fine-tuning the ethylene and abscisic acid signaling pathways. Collectively, our study demonstrates that SFB application can significantly enhance the fruit quality of ‘Shine-Muscat’ grapes and reveals its strong commercial potential as an eco-friendly alternative. However, several questions remain to be addressed in future investigations: the precise molecular targets of SFB-derived bioactive compounds need to be identified; the crosstalk between redox homeostasis and phytohormone signaling networks requires further exploration; and the dose- and time-dependent effects of SFB treatment under different storage conditions warrant systematic evaluation.

## 6. Future Perspectives

Future studies will focus on isolating and characterizing the active components in SFB, employing multi-omics approaches to elucidate the global regulatory networks, and validating these findings in other economically important horticultural crops to assess broader applicability. These investigations will not only advance the understanding of postharvest senescence mechanisms, but may also may lead to novel, eco-friendly preservation technologies, ultimately reducing food waste and improving producers’ economic returns.

## 7. Limitations and Challenges

The limitations and challenges of the current study include the following: the exclusive use of the ‘Shine Muscat’ cultivar with limited biological replicates constrains extrapolation to other *V. vinifera* varieties, controlled laboratory conditions may not accurately simulate dynamic commercial storage environments, and inherent batch-to-batch variability in SFB metabolite profiles persists despite rigorous standardization procedures.

## Data Availability

The original data presented in the study are openly available in the Sequence Read Archive (SRA) database under accession numbers SRX26798407-SRX26798415.

## Supplementary Materials

The following supporting information can be downloaded at: https://www.mdpi.com/article/10.3390/genes16040468/s1, Table S1: The detailed information of interaction network among the differentially expressed genes generated by String 12.0.; Table S2: Information of clusters generated by MCODE plugin.
