# Supplementary material for: Physiological and Transcriptomic Analyses Unveil the Preservation Mechanism of Streptomyces albulus Ah11601 Fermentation Broth on ‘Shine Muscat’ Grapes
Source: Genes (Basel). 2025 Apr 19;16(4):468. doi: 10.3390/genes16040468 (PMC12026805; doi:10.3390/genes16040468)
Supplement: Supplementary file 1 [file genes-16-00468-s001.zip › Table S1.pdf]

Table S1. The detailed information of interaction network among the differentially expressed genes generated by String 12.0.

**Node1**	**Node2**	**Neighborhood on chromosome**	**Phylogenetic cooccurrence**	**Homology**	**Coexpression**	**Experimentally determined interaction**	**Database annotated**	**Automated textmining**	**Combined score**
4CL	CCoAOMT	0	0	0	0.424	0.048	0.664	0.901	0.979
4CL	CHS	0.07	0	0	0.126	0	0.841	0.813	0.972
4CL	PAL	0.062	0	0	0.449	0	0.65	0.93	0.985
4CL	CHI	0	0	0	0.055	0	0	0.638	0.643
4CL	C4H	0	0	0	0.345	0.078	0.908	0.929	0.995
4CL	LDOX	0	0	0	0	0	0.106	0.801	0.814
4CL	F3'H	0	0	0	0.068	0.078	0.12	0.824	0.849
4CL	F3H	0	0	0	0.048	0	0.106	0.882	0.89
CCoAOMT	COMT	0	0	0	0	0	0	0.513	0.513
CCoAOMT	LDOX	0	0	0	0	0	0	0.738	0.738
CCoAOMT	F3'H	0	0	0	0.05	0	0	0.739	0.741
CCoAOMT	F3H	0	0	0	0.057	0	0	0.756	0.76
CCoAOMT	PAL	0	0	0	0.435	0	0	0.921	0.953
CCoAOMT	C4H	0	0	0	0.442	0	0	0.932	0.96
CCoAOMT	4CL	0	0	0	0.424	0.048	0.664	0.901	0.979
CCoAOMT	CHS	0	0	0	0.062	0	0.907	0.782	0.979
CHI	CHS	0	0	0	0.455	0.292	0.9	0.945	0.997
CHI	PAL	0	0	0	0.078	0	0	0.784	0.792
CHI	4CL	0	0	0	0.055	0	0	0.638	0.643
CHI	C4H	0	0	0	0.079	0	0	0.791	0.799
CHI	LDOX	0	0	0	0.078	0	0	0.881	0.885
CHI	F3'H	0	0	0	0.384	0	0.9	0.898	0.993

CHI	F3H	0	0	0	0.446	0.292	0.9	0.907	0.995
CHS	CCoAOMT	0	0	0	0.062	0	0.907	0.782	0.979
CHS	PAL	0	0	0	0.082	0	0	0.954	0.956
CHS	LDOX	0	0	0	0.099	0	0.104	0.967	0.971
CHS	4CL	0.07	0	0	0.126	0	0.841	0.813	0.972
CHS	F3'H	0.041	0	0	0.317	0.047	0.149	0.964	0.977
CHS	F3H	0	0	0	0.499	0.292	0.104	0.977	0.991
CHS	C4H	0.041	0	0	0.098	0.047	0.911	0.953	0.995
CHS	CHI	0	0	0	0.455	0.292	0.9	0.945	0.997
CKX3	IPT3	0	0	0	0	0	0	0.8	0.8
CKX3	IPT5	0	0	0	0	0	0	0.803	0.803
CKX3	CYP735A	0	0	0	0.054	0	0.062	0.757	0.765
CYP735A	IPT3	0	0	0	0.047	0	0.9	0.795	0.978
CYP735A	IPT5	0	0	0	0.069	0	0.9	0.785	0.978
CYP735A	CKX3	0	0	0	0.054	0	0.062	0.757	0.765
C4H	CCoAOMT	0	0	0	0.442	0	0	0.932	0.96
C4H	CHS	0.041	0	0	0.098	0.047	0.911	0.953	0.995
C4H	PAL	0	0	0	0.451	0	0.9	0.979	0.998
C4H	CHI	0	0	0	0.079	0	0	0.791	0.799
C4H	F3'H	0	0.152	0.771	0	0	0	0.628	0.671
C4H	LDOX	0	0	0	0	0	0	0.932	0.932
C4H	F3H	0	0	0	0.115	0	0	0.982	0.983
C4H	4CL	0	0	0	0.345	0.078	0.908	0.929	0.995
F3'H	CCoAOMT	0	0	0	0.05	0	0	0.739	0.741
F3'H	CHS	0.041	0	0	0.317	0.047	0.149	0.964	0.977

F3'H	PAL	0	0	0	0.049	0	0	0.949	0.949
F3'H	CHI	0	0	0	0.384	0	0.9	0.898	0.993
F3'H	C4H	0	0.152	0.771	0	0	0	0.628	0.671
F3'H	4CL	0	0	0	0.068	0.078	0.12	0.824	0.849
F3'H	LDOX	0	0	0	0.208	0	0.9	0.98	0.998
F3'H	F3H	0	0	0	0.308	0	0.9	0.987	0.999
EBF1	SnRK2A	0	0	0	0	0.474	0	0.543	0.749
EBF1	ETR2	0	0	0	0.045	0	0	0.861	0.861
EBF1	ERS1	0	0	0	0.07	0	0	0.863	0.867
EBF1	ERF1B	0	0	0	0.045	0	0	0.794	0.794
ERF1B	PAL	0	0	0	0	0	0	0.409	0.408
ERF1B	ETR2	0	0	0	0.081	0	0	0.76	0.77
ERF1B	ERS1	0	0	0	0.05	0	0	0.793	0.795
ERF1B	EBF1	0	0	0	0.045	0	0	0.794	0.794
ERS1	ETR2	0	0.118	0.837	0.21	0	0	0.551	0.66
ERS1	ERF1B	0	0	0	0.05	0	0	0.793	0.795
ERS1	EBF1	0	0	0	0.07	0	0	0.863	0.867
ETR2	ERS1	0	0.118	0.837	0.21	0	0	0.551	0.66
ETR2	ERF1B	0	0	0	0.081	0	0	0.76	0.77
ETR2	EBF1	0	0	0	0.045	0	0	0.861	0.861
F3H	CCoAOMT	0	0	0	0.057	0	0	0.756	0.76
F3H	CHS	0	0	0	0.499	0.292	0.104	0.977	0.991
F3H	PAL	0	0	0	0.116	0	0	0.932	0.937
F3H	CHI	0	0	0	0.446	0.292	0.9	0.907	0.995
F3H	C4H	0	0	0	0.115	0	0	0.982	0.983

F3H	4CL	0	0	0	0.048	0	0.106	0.882	0.89
F3H	LDOX	0	0.145	0.784	0.174	0	0.9	0.621	0.969
F3H	COMT	0	0	0	0	0	0	0.437	0.437
F3H	F3'H	0	0	0	0.308	0	0.9	0.987	0.999
HPPD	PAL	0.109	0	0	0.226	0	0	0.367	0.526
HPPD	PAT	0	0	0	0.123	0	0.938	0.194	0.952
HSP15.7	HSP17.4	0	0.094	0.883	0.305	0	0	0.129	0.404
HSP15.7	HSP22.0	0	0.118	0.83	0.398	0	0	0.168	0.52
HSP15.7	HSP23.6	0	0.146	0.766	0.308	0	0	0.268	0.53
HSP17.4	HSP15.7	0	0.094	0.883	0.305	0	0	0.129	0.404
HSP17.4	HSP22.0	0	0.091	0.897	0.366	0	0	0.119	0.448
HSP17.4	HSP70-2	0.051	0	0	0.254	0.049	0	0.296	0.462
HSP17.4	HSP23.6	0	0.141	0.748	0.449	0	0	0.238	0.607
HSP18.1	HSP70-1	0.051	0	0	0.254	0.049	0	0.295	0.462
HSP18.1	HSP23.6	0	0.144	0.736	0.256	0.089	0	0.24	0.5
HSP18.1	HSP22.0	0	0.09	0.9	0.451	0	0	0.127	0.526
HSP22.0	HSP17.4	0	0.091	0.897	0.366	0	0	0.119	0.448
HSP22.0	HSP18.1	0	0.09	0.9	0.451	0	0	0.127	0.526
HSP22.0	HSP70-1	0.051	0	0	0.254	0.049	0	0.245	0.424
HSP22.0	HSP15.7	0	0.118	0.83	0.398	0	0	0.168	0.52
HSP22.0	HSP23.6	0	0.451	0	0.355	0	0	0.876	0.952
HSP23.6	HSP17.4	0	0.141	0.748	0.449	0	0	0.238	0.607
HSP23.6	HSP18.1	0	0.144	0.736	0.256	0.089	0	0.24	0.5
HSP23.6	HSP70-1	0.051	0	0	0.254	0.049	0	0.217	0.402
HSP23.6	HSP22.0	0	0.451	0	0.355	0	0	0.876	0.952

HSP23.6	HSP15.7	0	0.146	0.766	0.308	0	0	0.268	0.53
HSP70-1	HSP18.1	0.051	0	0	0.254	0.049	0	0.295	0.462
HSP70-1	HSP23.6	0.051	0	0	0.254	0.049	0	0.217	0.402
HSP70-1	HSP22.0	0.051	0	0	0.254	0.049	0	0.245	0.424
HSP70-2	HSP17.4	0.051	0	0	0.254	0.049	0	0.296	0.462
IPT3	CKX3	0	0	0	0	0	0	0.8	0.8
IPT3	CYP735A	0	0	0	0.047	0	0.9	0.795	0.978
IPT5	CKX3	0	0	0	0	0	0	0.803	0.803
IPT5	CYP735A	0	0	0	0.069	0	0.9	0.785	0.978
LDOX	CCoAOMT	0	0	0	0	0	0	0.738	0.738
LDOX	CHS	0	0	0	0.099	0	0.104	0.967	0.971
LDOX	PAL	0	0	0	0	0	0	0.941	0.941
LDOX	CHI	0	0	0	0.078	0	0	0.881	0.885
LDOX	C4H	0	0	0	0	0	0	0.932	0.932
LDOX	4CL	0	0	0	0	0	0.106	0.801	0.814
LDOX	F3H	0	0.145	0.784	0.174	0	0.9	0.621	0.969
LDOX	F3'H	0	0	0	0.208	0	0.9	0.98	0.998
PAL	CCoAOMT	0	0	0	0.435	0	0	0.921	0.953
PAL	CHS	0	0	0	0.082	0	0	0.954	0.956
PAL	ERF1B	0	0	0	0	0	0	0.409	0.408
PAL	HPPD	0.109	0	0	0.226	0	0	0.367	0.526
PAL	PAT	0	0	0	0.179	0	0.65	0.202	0.751
PAL	CHI	0	0	0	0.078	0	0	0.784	0.792
PAL	F3H	0	0	0	0.116	0	0	0.932	0.937
PAL	LDOX	0	0	0	0	0	0	0.941	0.941

PAL	F3'H	0	0	0	0.049	0	0	0.949	0.949
PAL	4CL	0.062	0	0	0.449	0	0.65	0.93	0.985
PAL	C4H	0	0	0	0.451	0	0.9	0.979	0.998
PAT	PAL	0	0	0	0.179	0	0.65	0.202	0.751
PAT	HPPD	0	0	0	0.123	0	0.938	0.194	0.952
PYR1	SnRK2A	0	0	0	0	0	0	0.739	0.739
SnRK2A	PYR1	0	0	0	0	0	0	0.739	0.739
SnRK2A	EBF1	0	0	0	0	0.474	0	0.543	0.749
COMT	CCoAOMT	0	0	0	0	0	0	0.513	0.513
COMT	F3H	0	0	0	0	0	0	0.437	0.437
