# Supplementary material for: Physiological and Transcriptomic Analyses Unveil the Preservation Mechanism of Streptomyces albulus Ah11601 Fermentation Broth on ‘Shine Muscat’ Grapes
Source: Genes (Basel). 2025 Apr 19;16(4):468. doi: 10.3390/genes16040468 (PMC12026805; doi:10.3390/genes16040468)
Supplement: Supplementary file 1 [file genes-16-00468-s001.zip › Table S2.pdf]

Table S2. Information of clusters generated by MCODE plugin.

Cluster	Score	Node	Edge	Seed node	Node name
1	8.75	9	70	CCoAOMT	LDOX, 4CL, C4H, PAL, CHI, F3H, F3'H, CCoAOMT, CHS
2	4	4	12	EBF1	ERF1B, EBF1, ERS1, ETR2
3	3.33	4	10	IPT3	CYP735A, IPT5, IPT3, CKX3
